# A decade of plague in Madagascar: a description of two hotspot districts

**DOI:** 10.1186/s12889-021-11061-8

**Published:** 2021-06-10

**Authors:** Sitraka Rakotosamimanana, Daouda Kassie, François Taglioni, Josélyne Ramamonjisoa, Fanjasoa Rakotomanana, Minoarisoa Rajerison

**Affiliations:** 1 Institut Pasteur de Madagascar, Antananarivo, Madagascar; 2 Université d’Antananarivo, Antananarivo, Madagascar; 3 Université de La Réunion, La Réunion, France; 4 Centre de Coopération Internationale en Recherche Agronomique pour le Développement, CIRAD UMR ASTRE, Antananarivo, Madagascar; 5 ASTRE, Université de Montpellier, CIRAD, INRAE, Montpellier, France

**Keywords:** Plague, Madagascar, Central highlands, Epidemiology, Deviation from the decadal mean of the incidence (DDMI)

## Abstract

**Background:**

Human plague cases, mainly in the bubonic form, occur annually in endemic regions of the central highlands of Madagascar. The aim of this study was to compare the dynamics of the epidemiological features of the human plague in two districts of the central highlands region.

**Methods:**

In Madagascar, all clinically suspected plague cases that meet clinical and epidemiological criteria specified in the World Health Organization (WHO) standard case definition are reported to the national surveillance system. Data on plague cases reported between 2006 and 2015 in the districts of Ambositra and Tsiroanomandidy were analysed. Statistical comparisons between the epidemiological characteristics of the two districts were conducted.

**Results:**

A total of 840 cases of plague were reported over the studied period, including 563 (67%) probable and confirmed cases (P + C). Out of these P + C cases, nearly 86% (488/563) were cases of bubonic plague. Reported clinical forms of plague were significantly different between the districts from 2006 to 2015 (*p = 0.001*). Plague cases occurred annually in a period of 10 years in the Tsiroanomandidy district. During the same period, the Ambositra district was characterized by a one-year absence of cases.

**Conclusion:**

The differences in the epidemiological situation with respect to the plague from 2006 to 2015 in the two central highlands districts may suggest that several factors other than biogeographical factors determine the representation of the plague and its dynamics in this region. Considering the epidemiological situations according to the specific contexts of the districts could improve the results in the fight against the plague in Madagascar.

## Background

Plague is a zoonotic disease caused by *Yersinia pestis* bacteria. Humans can be infected after being bitten by fleas from rats infected by *Y. pestis* and then develop bubonic plague, which can evolve into other forms of plague, pneumonic plague and septicaemic plague. The disease can be treated with antibiotics, but delays in treatment can result in death or the evolution of the disease to the pneumonic or septicaemic forms [1, 2].

Plague is a notifiable disease. Worldwide, since the 2000s, cases of human plague have occurred on three main continents: Asia, America and Africa [3, 4]. Africa was the most affected continent in the world between 2013 and 2018, accounting for 96.7% (2791/2886) of the global cases. The majority of the notified global cases, i.e., a total of 80.5% (2323/2886) of the cases during this period, were reported in Madagascar [3]. Plague is a public health concern in Madagascar [4–6]. Every year, approximately 400 cases of human plague are reported in this country [4]. The predominant form of human plague in Madagascar is the bubonic plague, but cases of pneumonic plague are also reported [5–10]. Outbreaks of pneumonic plague may also occur in Madagascar, as recently reported in 2017, with 2417 cases and 209 deaths, representing a case-fatality rate of 8.6% [11, 12].

Since 1898, when the first cases of human plague appeared in Madagascar, most cases have appeared in the central highlands region. The plague is endemic in this region which is located at an altitude of more than 800 m [7, 8, 10]. The plague season is generally between October and April in this region, where cases appear almost every year, with intraregional heterogeneities, but some cases are reported outside this period [9]. Case reporting may be annual in the central highlands, but in some cases, periods of inactivity (absence of cases) may characterize the years. However, the disease can also appear in coastal areas [7–8].

As the plague remains a major public health concern in Madagascar, this study was conducted to contribute to a better understanding of the dynamic of plague and the control of this disease in endemic areas. The aim of this study is to (1) describe the epidemiological characteristics and dynamics of the human plague in two districts of the central highlands between 2006 and 2015 and (2) compare the epidemiological situations of the human plague in these two districts during the same time period.

## Methods

### Study areas

**Fig. 1:**
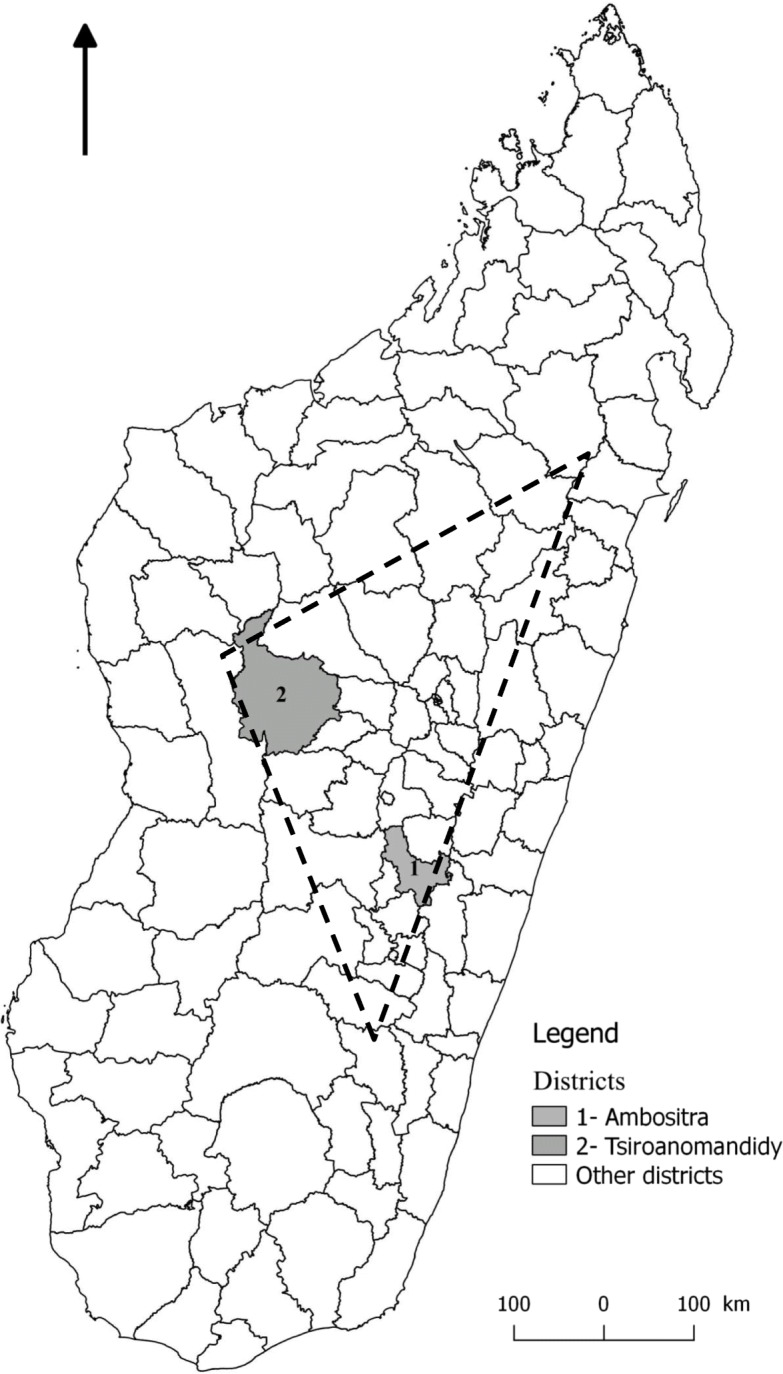
Locations of the studied districts and the plague triangle - The map was performed by S. Rakotosamimanana using free and open source Quantum GIS (QGIS) 3.4® software

The district of Ambositra is located in the southern central highlands and includes 23 municipalities subdivided into 290 *fokontany* (the smallest administrative unit in Madagascar) and covers 3161 km^2^. The district of Tsiroanomandidy is located in the middle west of the central highlands; it has 18 municipalities subdivided into 212 *fokontany* and covers 10,199 km^2^. Both districts are part of the geographical triangle of the plague endemic districts (Fig. 1) in the central highlands or the “plague triangle” [13, 14].

These two districts were chosen because they belong to the central highlands region and because the Ambositra district had a very active endemic plague outbreak in the 2000s [10] and the Tsiroanomandidy district is presently one of the main active plague outbreak regions [15].

### Study design

This study is based on a retrospective and comparative descriptive analysis of data on reported plague cases between 2006 and 2015 in two districts in the central highlands of Madagascar.

### The database

As plague is a notifiable disease, according to the National Plague Control Program (NPCP), all data relating to the reporting of plague cases in Madagascar are centralized in a database at the Central Plague Laboratory (CPL), which is managed by the *Institut Pasteur de Madagascar*/Ministry of Public Health. The database contains plague cases declared in health facilities between 1 January 2006 and 31 December 2015 for the two districts in the central highlands. The database contains various information including information on individuals such as address, age, sex, reference health facility where the case was reported, onset date of the disease (day/month/year), clinical form of plague (bubonic, pneumonic, septicaemic, etc.), patient status (alive or deceased), travel made before the case was reported (yes/no), presence of dead rats observed in the vicinity of the reported case (yes/no), case definition according to the type of tests performed and other criteria (suspect, probable, confirmed).

A total of 840 plague cases were reported for the two districts between 2006 and 2015.

### Case definition

According to Madagascar’s Ministry of Public Health and the World Health Organization (WHO), plague cases are defined in three categories based on clinical and epidemiological criteria and the results of tests performed on biological samples [4, 10]:

- Suspected case (S): all persons presenting clinical signs suggestive of plague in a favourable epidemiological context.
- Probable case (P): any suspected person with a positive result for one of the following tests, F1 rapid diagnostic test (RDT), serological test or PCR, and where isolation of *Y. pestis* by culture has not been performed or is negative.
- Confirmed case (C): any suspected person for whom an isolation of *Y. pestis* by culture has been performed or a conversion observed or positive RDT and PCR.

### Temporal trend of plague incidence during the study period in both districts

A time series analysis was performed based on the deviation from the decadal mean of the incidence (DDMI) for a given month. DDMI is the difference between monthly incidences compared to monthly decadal mean incidences of plague cases during the study period.

Based on demographic data from the RGPH 2018 (the last official census in Madagascar dates back to 1993), we estimated the number of people in both districts between 2006 and 2015 (for an annual population growth rate of 2.9% in Madagascar). We calculated the monthly mean incidence (number of cases per 100,000 inhabitants) and the mean of human cases (P + C) of plague between the studied periods for each district. We then estimated the incidence per month for each year of the study period and divided the study period into 120 months or 10 years. Then, we calculated the monthly decadal mean incidence.

The DDMI trend was analysed by district with a linear regression line for the studied period. The expected DDMI *y* of a given month is determined by the linear regression line defined by the formula *y = ax + b,* where.

*y* = expected DDMI for a given month,

*x* = previous month’s DDMI,

*a* = steering coefficient of the line (constant) and.

*b* = the intercept (constant).

To detect seasonality in the monthly DDMI, we divided the time series into four-month seasons by calculating moving averages.

### Data processing and analysis

Processing, database management and statistical analyses were carried out using Microsoft Excel® and Stata 13® software. Qualitative variables are represented as frequencies and/or proportions. For the comparisons between the two districts, we used the chi-squared test of comparison or Fisher’s exact test, if appropriate, for the qualitative variables. For the comparison of the quantitative variables between the two districts, we used Student’s t-test for independent samples. The significance threshold was set at *p < 0.05*.

## Results

### Characteristics of overall reported plague cases in both districts

**Fig. 2:**
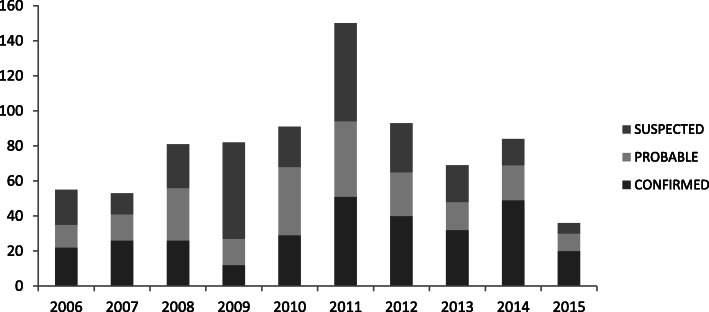
Suspected, probable and confirmed cases reported annually in the two districts from 2006 to 2015

Out of the 840 cases reported in the two districts for the period under review, approximately 33% (277/840) were suspected cases (S), 28.4% (239/840) were probable cases (P) and 38.6% (324/840) were confirmed cases (C) (Fig. 2).

In terms of demographic characteristics, approximately 41% (341/840) of the cases were females. The age of the individuals was recorded in the database for a 98.7% (829/840) of the cases. The median age of reported cases was 12 years old.

Out of the 563 probable and confirmed cases (P + C), 4.4% (25/563) were cases of pneumonic plague (PP), and approximately 91% (512/563) were cases of bubonic plague (BP). Cases with an unreported clinical form represented 4.6% (26/563) of the reported P + C cases.

Out of the 840 reported cases, 838 cases had information on dead rat presence or absence in the vicinity of the reported case dwellings. Reports of dead rats accounted for almost 18% (150/838) of the reported cases. The reports of dead rats in Tsiroanomandidy district were significantly higher than that in Ambositra district (19.9% vs. 9.5%; *p = 0.002*).

### Age range and sex among P + C cases in both districts

For both districts, out of the 563 P + C cases, the ages of 558 individuals were reported. Nearly 41% of the individuals were female (227/558). The most represented age group was 10–19 years old, which accounted for 35.8% (200/558) of the total P + C cases for the study period. Approximately 69% (387/558) of the P + C cases were < 19 years old, and just over 1% (7/558) were over 60 years old. The demographic and epidemiological characteristics of the reported P + C cases by district are reported in Table 1.

**Table 1:** Demographic and epidemiological characteristics of the reported P + C cases by district

Features/Districts	Ambositra	Tsiroanomandidy
P + C (N)	105	458
Sex-ratio (Male/Female)	1.14 (56/49)	1.58 (279/179)
Age median (25–75 percentiles)	13 (8–36)	12 (8–22)

### Age and sex of P + C cases by district

**Fig. 3:**
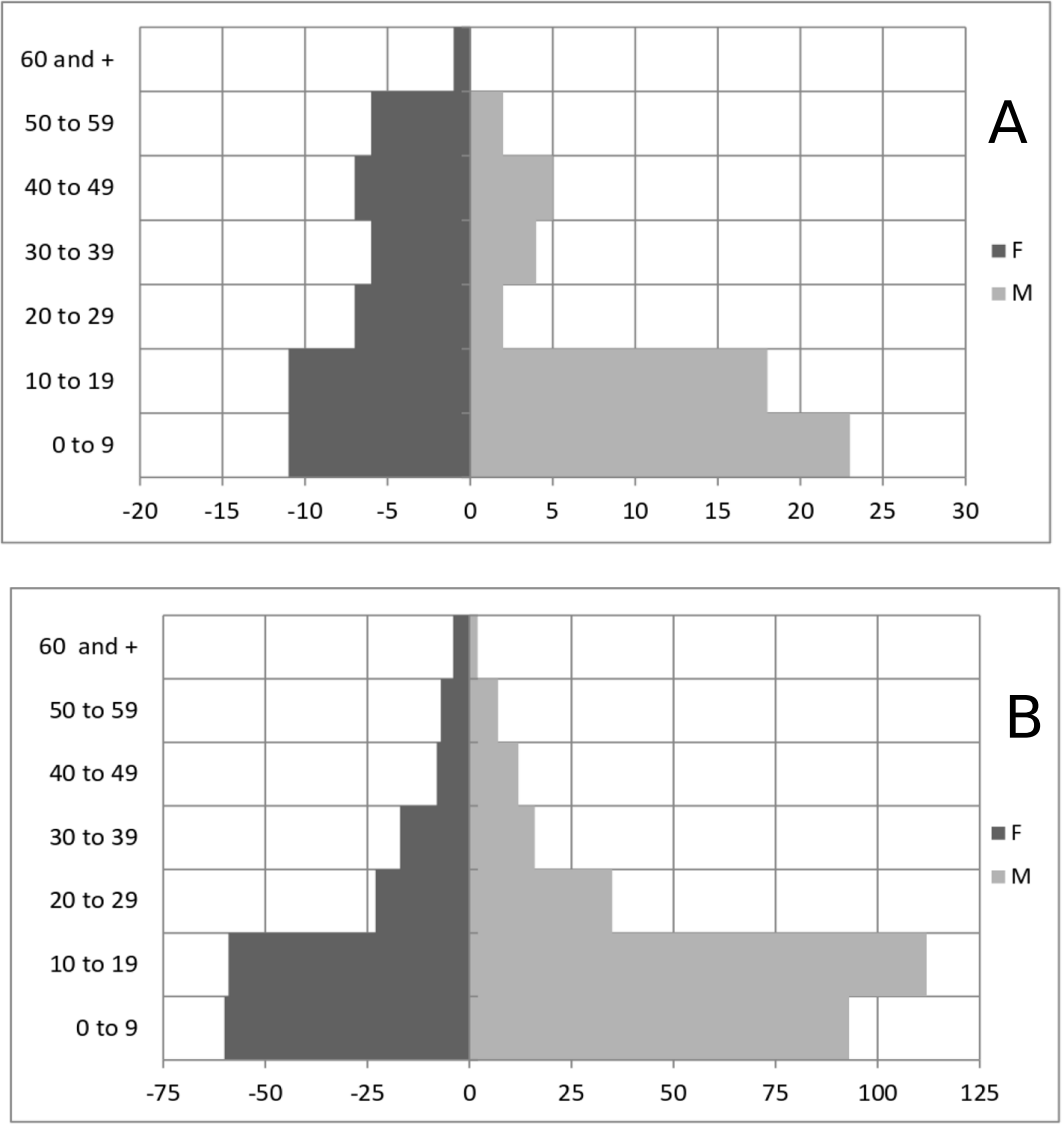
Confirmed and probable plague cases by sex and 10-year age groups in Ambositra (**A**) and Tsiroanomandidy (**B**), Madagascar, 2006–2015

For the Ambositra district, ages were reported for all 103 P + C cases, and just over 47% (49/103) of the individuals were female. Individuals under 9 years old accounted for approximately 33% (34/103) of the total P + C cases in this district. Individuals less than 19 years old accounted for almost 61% (63/103) of the total P + C cases reported during the study period (Fig. 3A). Approximately 1% (1/103) of the P + C cases were at least 60 years old.

Concerning the district of Tsiroanomandidy, out of the 458 P + C cases reported between 2006 and 2015, 455 cases had age information in the database. Almost 39% (178/455) of the individuals were female. Individuals under 19 years old accounted for approximately 71% (324/455) of the total P + C cases during the study period. In addition, 37.5% (171/455) of the total P + C cases were between 10 and 19 years old. Just over 1% of the P + C (6/455) were at least 60 years old (Fig. 3B).

There were no significant sex differences among the P + C cases in either district during the study period.

### P + C clinical form by district

For the district of Ambositra, approximately 81% (85/105) of the P + C cases were BP cases, and 9.5% (10/105) were PP cases. 9.5% (10/105) were undocumented clinical cases of plague (Table 2).

**Table 2:** P + C cases per clinical form in both districts during the studied period

District/Clinical form	BP		PP		Undocumented		Total	
	n	%	n	%	n	%	n	%
Ambositra	85	81	10	9.5	10	9.5	105	100
Tsiroanomandidy	427	93.2	11	2.4	20	4.4	458	100
Both districts	512	91	21	3.7	30	5.3	563	100

For Tsiroanomandidy, 93.2% (427/458) of the P + C cases were BP cases, 2.4% (11/458) were PP cases and 4.4% (20/458) were clinically undocumented plague cases (Table 2).

The clinical forms (BP and PP) of the P + C cases were significantly different between the two districts (*p < 0.001*) for the 533 cases with reported clinical forms during the study period (Table 2).

### Annual cases reported by district

For both districts, the number of cases varied from year to year for the studied period.

From 2006 to 2015 in the Ambositra district, 18 out of 23 municipalities reported the occurrence of P + C plague cases. A peak of probable and confirmed cases was reported for 2007 with 29 cases (28 cases of BP and 1 case of PP), representing approximately 28% of the P + C cases that were reported in this district from 2006 to 2015. The year 2010 was a period of silence in this district, as no P + C cases of plague were reported; however, one suspected case was reported in that year.

For the district of Tsiroanomandidy, 16 out of 17 municipalities reported the occurrence of P + C cases during the period under review. The year 2011 was a peak year of P + C cases, with 97 cases (89 PB cases, 3 PP cases and 5 undocumented cases), representing approximately 21% of the P + C cases that were reported during the studied period. The lowest number of P + C cases was reported in 2007, with 12 reported cases (approximately 3% of the total P + C cases from 2006 to 2015 in this district), all of which were BP.

### Temporal trend of DDMI of plague cases in both districts for the studied period

For the district of Ambositra, no plague cases were reported in July in any year of the period under study. A period of silence marked the year 2010 before a resumption of plague activity during the last quarter of 2011. Non-significant changes in the incidence were detected for February (year 2013), March (years 2014 and 2015), July (for the entire studied period), and October (years 2007, 2008, 2013, and 2014 to 2015).

**Fig. 4:**
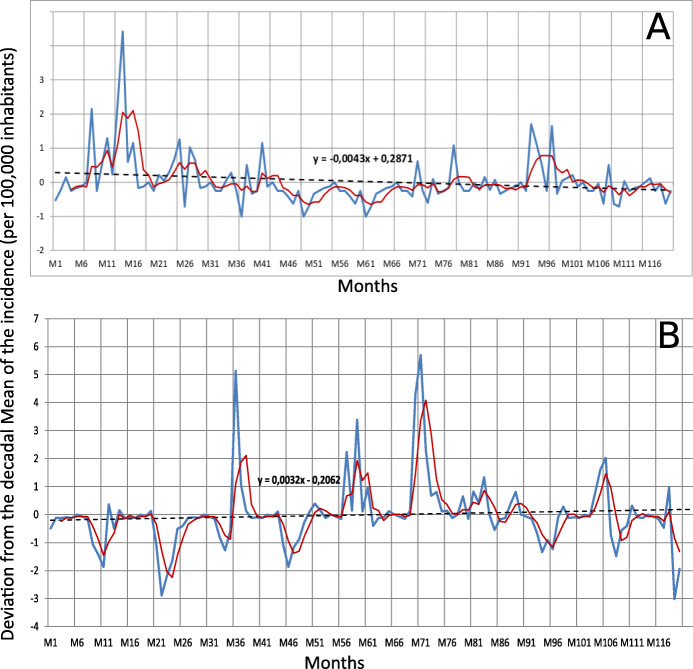
Temporal trends in DDMI of plague cases from month 1 to month 120 in the districts of Ambositra (**A**) and Tsiroanomandidy (**B**) - Legend. The blue curve represents the DDMI; the red curve represents the quarterly moving averages of the DDMI; the dashed line is a linear regression line showing the trend of the DDMI for the period under study

From 2006 to 2015, a downward trend in the DDMI of plague cases was detected in the Ambositra district (Fig. 4A). The expected DDMI for a given month can be determined by the equation *y = − 0.0043x + 0.2871* for this district.

For Tsiroanomandidy district in the studied period, the plague season generally begins between July and September each year before reaching a peak between October and December. No plague cases were reported during June in any year of the period under study. In this district for the entire studied period, DDMI without major variations was detected during April (for the years 2006 to 2009 and 2011), June (for the entire study period) and December (year 2012).

From 2006 to 2015, a slight upward trend in the DDMI of plague cases was detected in the district of Tsiroanomandidy during the study period. The DDMI for a given month for Tsiroanomandidy district can be determined by the equation *y = 0.0032x - 0.2062* (Fig. 4B).

Division into four-month seasons showed irregularity in the DDMI for the entire period under study in both districts.

The DDMI in both districts did not follow the same trend during the same period.

## Discussion

Although the first cases of human plague appeared in Madagascar in 1898, the plague still affects hundreds of people annually, particularly in the central highlands where it is endemic. We conducted a comparative descriptive study by analysing epidemiological data on the plague in two endemic districts in the central highlands of Madagascar for a period of 10 years. The results of the study showed differences in the epidemiological characteristics of the dynamics of the human plague in the two districts during the studied period.

For the two studied districts during the studied decade, nearly 67% of the plague cases were either probable or confirmed (P + C). This is higher than the amount of P + C cases reported in other countries, for instance, in Uganda between 2008 and 2016, in which P + C cases represented 46% of the total cases [16]. For the case of Madagascar between 1998 and 2016, P + C cases accounted for 44% of the total reported human plague cases. This difference in P + C proportions could be explained by the fact that the two districts studied were hotspots of human plague in Madagascar during the studied period [17]. This suggests that despite the absence of plague cases in several districts of Madagascar, some districts influence the national average. The endemic districts in our study had a higher proportion of plague cases than the national average between 1998 and 2016 [17]. The proportion of P + C cases in both districts was nearly 33% of the total reported cases.

Most P + C cases reported in both districts were in individuals under 19 years old, in which is similar to most countries in Sub-Saharan Africa [16, 18, 19], where the human plague is rampant. This characteristic has also been observed in epidemiological studies of the plague in Madagascar [12–17] and worldwide [20]. This can be explained by the sociocultural practices and behaviours of individuals in this age group. Indeed, several authors explain this fact as a difference in the exposure of these individuals to the risk of plague for different age categories and according to sex, especially in rural areas due to agricultural activities [20]. As the rural areas of the central highlands are often areas with strong agricultural practices, individuals under 19 years old are more likely to be required to work in the fields than individuals in other age groups [21, 22]. These facts have also been observed in areas of plague endemicity in Sub-Saharan Africa [23, 24].

Regarding clinical forms, bubonic plague (BP) cases represented 91% of the P + C cases in both districts for the studied period. Pneumonic plague (PP) represented 4% of the P + C cases for the same period. These proportions are different from the BP cases recorded, for instance, in the Democratic Republic of Congo (DRC). In the DRC, BP and PP represented 79 and 9%, respectively, of the reported cases between 2004 and 2014 [25]. However, the proportions of BP in the two districts were not different from the proportion of BP in Madagascar between 1998 and 2016. This national proportion of 90% is also the average proportion of BP in Zimbabwe according to a study conducted between 1974 and 2018 [26]. At the level of the two studied districts, according to our results, there was a significant difference in the clinical cases reported during the studied time period. This suggests that among the populations of two different districts in the central highlands, the disease does not evolve in the same way. This may suggest a difference in the behavioural factors of the population towards the plague between the two districts. There was a higher proportion of PP cases in the Ambositra district than in the Tsiroanomandidy district. This can be explained by the fact that the behaviour of plague patients is probably different between the two districts. This could influence the delays in patient management and would promote the multiplication of BP cases that evolve to PP in the Ambositra district for the studied period.

These varied situations in the epidemiological expression of the human plague for two districts in the central highlands with an average altitude of over 800 m could also be explained by the different biogeographical characteristics between these two districts. Indeed, although these districts are in a region known as the central highlands, these two districts are different both in terms of climate and the vegetation found in them. As the plague is primarily a zoonosis, human exposure to the plague is dependent on environmental and climatic factors that bring infected rats and their fleas into contact with humans [24, 27–30]. Socio-political circumstances could also have contributed to the under-reporting of plague cases in Madagascar [31]. The absence of cases in Ambositra in 2009 can be explained by the development of a socio-political crisis in Madagascar. To this end, the difficulties caused by the lack of logistics or materials and the geographical remoteness or landlocking of certain areas can be added to this observation, making it impossible to send biological samples to the Central Plague Laboratory located at the capital [10].

The deviation from the decadal mean of the incidence (DDMI) of plague cases did not vary in the same way in the two central highland districts between 2006 and 2015. The DDMI trend in the Tsiroanomandidy district increased slightly, while the trend decreased slightly in the Ambositra district. The peak plague season usually began in July and August in the Tsiroanomandidy district during the studied period, while in the Ambositra district, the high plague season varied from year to year. During the studied period, the Ambositra district had a year in which there were no reported plague cases (silent period), while the Tsiroanomandidy district reported plague cases in all of the studied years. The DDMIs for both districts were irregular and varied from year to year. These differences in context and trends in DDMI can be explained by environmental-climatic factors (El Niño phenomenon). This fact was suggested by Kreppel and her team, who highlighted relationships between climatic oscillations due to ENSO (El Niño Southern Oscillation) phenomena and the incidence of human plague in Madagascar [32]. ENSO can affect the microclimate of the central highlands region differently and thus impacts the occurrences of human plague cases. Very few or no studies on the impact of other factors on plague incidence have been carried out in Madagascar.

Apart from this, the differences in the epidemiological context in relation to the human plague in the two central highlands districts could also be explained by the geographical characteristics of the two districts. The Tsiroanomandidy district is larger in area than the Ambositra district. This fact may have consequences for the management of human plague epidemics because the territory is large. The Ambositra district is facilitated by the proximity of the health infrastructure to the population because it is a more densely populated and smaller district than Tsiroanomandidy. On the other hand, in Tsiroanomandidy, the surface area is larger and the district is less densely populated, the health structures are located further from the population. The spatial control in campaigns to combat the hosts and vectors of the plague can also be a determining factor in the district of Ambositra because of its area. However, the limited knowledge of the plague of the population in the two districts may play a role in the non-reporting of plague cases. Nevertheless, in theory, people living in plague endemic areas could easily recognize signs of the plague [28].

## Conclusion

The epidemiological characteristics of the human plague in the two central highlands districts suggest a new approach to plague control. Epidemiological contexts vary between districts in the same endemic area. It is difficult to eradicate plague or other diseases if efforts to control them are often neglected or not sustained. Intervention strategies should be adapted to the local and cultural specificities of the studied areas for plague and other diseases. Thus, taking into account the particular biogeographical, sociocultural and behavioural contexts of the different districts where the plague is endemic could improve the results in the fight against this disease in Madagascar. For instance, people living in areas where the human plague is endemic should be able to easily recognize signs of the plague if they have enough knowledge about these signs. This could be improved by the existence of frequent awareness campaigns in endemic areas. These campaigns will need to be adapted according to the population’s knowledge of the plague. Indeed, we conducted a knowledge, attitudes and practices (KAP) study [33] on the plague among the populations of these two districts (Ambositra and Tsiroanomandidy). This KAP article showed that people’s knowledge of the plague is heterogeneous according to the districts.

Moreover, establishing decentralized management of plague control in the allocation of material, human and financial resources according to the real needs of health administrations (depending on the real epidemiological, sociodemographic and sociocultural contexts) would contribute to an improvement in the plague situation at different administrative levels in Madagascar. Other studies on the links between human behaviour and the persistence of the plague in Madagascar could also provide more insight into the dynamics of the disease on the island and into similar diseases worldwide.

## Data Availability

The datasets generated and/or analysed during the current study are not publicly available but are available upon request to the corresponding author.

## Abbreviations

BP: Bubonic Plague
DDMI: Deviation from the Decadal Mean of the Incidence
ENSO: El Niño Southern Oscillation
KAP: Knowledge, Attitudes and Practices
PP: Pneumonic Plague
SR: Sex Ratio
WHO: World Health Organization
